# Supplementary material for: Hemodynamic effects of high frequency oscillatory ventilation with volume guarantee in a piglet model of respiratory distress syndrome
Source: PLoS One. 2021 Feb 16;16(2):e0246996. doi: 10.1371/journal.pone.0246996 (PMC7886162; doi:10.1371/journal.pone.0246996)
Supplement: S1 Table — (PDF) [file pone.0246996.s002.pdf]

Wt (kg)	1.7	1.9	1.7	1.7	1.7	1.9	1.7	1.9
Age (days)	2	3			2	2	2	3
Gender (1=M,	1	2	2	1	2	1	1	1
Treatment (1	HFOV	HFOV	HFOV	HFOV	HFOV	HFOV	HFOV	HFOV
Surgical time (	75	80	75	70	85	55	65	60
Lavage time (r	60	60	50	55	55	40	50	60
Lavage numb	9	13	11	11	11	8	10	13
HR (bpm)								
Post-surgery	243	220	213	208	292	288	158	186
Stable baseli	203	217	238	256	172	222	169	229
0	216	221	222	236	184	210	146	221
60	221	254	199	178	233	234	222	252
120	222	194	208	182	277	194	215	226
180	191	175	265	156	256	223	217	227
240	201	214	194	190	252	250	205	226
Mean bp (mmHg)								
Post-surgery	67	73	52	61	79	80	67	69
Stable baseli	74	74	56	73	71	62	65	66
0	98	67	57	68	64	63	52	70
60	93	67	44	51	75	73	80	69
120	102	44	53	51	61	64	57	62
180	62	42	72	37	71	63	56	50
240	58	56	37	40	52	57	60	39
Systolic bp (mmHg)								
Post-surgery	86	90	72	83	96	99	107	98
Stable baseli	91	107	80	106	98	91	109	95
0	124	92	79	95	93	88	84	92
60	115	97	64	67	109	94	108	94
120	125	71	71	75	87	73	85	83
180	75	50	92	51	102	76	83	77
240	74	88	58	55	82	71	96	64
Diastolic bp (mmHg)								
Post-surgery	53	58	39	45	66	67	46	50
Stable baseli	66	55	37	53	51	43	44	48
0	82	50	42	50	46	48	37	58
60	78	48	33	37	51	59	68	58
120	94	29	41	35	45	56	44	49
180	52	35	59	25	49	53	42	35
240	48	35	27	28	37	48	44	36

CO (ml/kg/min)

Stable baseli	82.9411765	83.6842105	124.7059	102.352941	77.6470588	126.842105	80.5882353	104.736842
0	60.5882353	116.842105	97.64706	76.4705882	51.7647059	85.7894737	60	38.4210526
60	53.5294118	147.894737	86.47059	59.4117647	77.0588235	58.9473684	98.2352941	65.7894737
120	47.0588235	107.894737	66.47059	88.8235294	78.8235294	73.6842105	68.2352941	70
180	68.2352941	101.052632	84.11765	87.6470588	125.294118	87.3684211	118.235294	74.2105263
240	114.117647	117.368421	62.94118	82.9411765	115.882353	102.631579	98.2352941	72.6315789

NIRS - cerebral (% saturation)

Post-surgery	47	40	43	47	45	48	33	48
Stable baseli	40	36	41	51	40	35	39	44
0	50	40	37	52	35	38	32	42
60	58	58	39	50	40	51	37	41
120	58	39	39	39	45	42	36	36
180	58	42	36	29	47	51	38	39
240	56	51	28	32	44	52	44	34

CA flow (ml/kg/min)

Post-surgery	60	55.7894737	31.76471	34.7058824	55.8823529	55.7894737	1.17647059	43.6842105
Stable baseli	40.5882353	35.2631579	52.35294	29.4117647	40	37.8947368	29.4117647	51.5789474
0	33.5294118	40.5263158	20	31.7647059	32.9411765	30	21.1764706	30
60	32.9411765	na	32.35294	18.23529412	30.5882353	31.0526316	18.8235294	17.3684211
120	40	na	31.17647	16.4705882	50	36.3157895	21.7647059	20
180	na	14.7368421	29.41176	9.41176471	54.1176471	na	27.6470588	13.6842105
240	30	11.0526316	22.35294	10.5882353	46.47058824	71.0526316	30.5882353	6.84210526

Lactate (mmol/L)

Post-surgery			3.01	4.28				
Stable baseli	2.36	3.46	3.49	4.03	4.11	3.61	4.15	6.63
0	2.29	3.88	3.78	4	3.67	3.14	4.11	7.88
60	3.13	na	4.94	2.95	3.13	2.85	3.44	8.35
120	na	na	5.51	3.6	na	4.16	na	na
180	3.33	na	na	4.33	na	4.38	na	na
240	3.03	na	6.22	2.99	1.8	4.36	2.16	12.03

SaO2 (%)

Post-surgery	94	95.5	98.8	94.5	92.1	95.1	98	96.4
Stable baseli	86	91.2	97.4	91.9	96.5	90.5	97.5	99.8
0	99.3	88.2	92.8	93.6	92.3	96.9	94.8	100
60	100	94.3	96.3	97.4	100	98.9	100	97.7

120	100	97.6	93.3	94.2	95.2	98.3	98.1	92.7
180	100	94.5	85.6	97.1	92.1	88.1	95.8	86.8
240	100	94.8	88.7	91	90.4	89.4	95.1	87.5
Paw (cmH2O)								
Post-surgery	10.8	9.6	9	9.3	9.8	9.4	8.7	10.5
Stable baseli	9.7	10.3	8.8	9.4	9.8	10.2	9.6	9.5
0	17	14.6	13.6	13	17.3	17.3	15	15
60	14	20	13	14.7	13	16.1	12.9	17.4
120	12	20	12	11.5	11.6	12	11.5	17.2
180	12	18	12	11.6	12	12	11.5	16.1
240	12	18	11.6	12	11.9	12	11.6	15.5
PEEP (cm H2O)								
Post-surgery	5.4	5.6	5.6	5.7	5.6	5.5	5.5	6.4
Stable baseli	5.6	5.4	6	5.8	5.5	5.4	5.4	5.5
0	na	hfo	7.6	hfo	hfo	hfo	hfo	hfo
60			hfo					
120								
180								
240								
PIP (cm H2O)								
Post-surgery	23	23	20	21	20	22	17	20
Stable baseli	23	25	22	22	26	23	21	20
0		hfo	31	hfo	hfo	hfo	hfo	hfo
60			hf					
120								
180								
240								
FiO2								
Post-surgery	0.23	0.25	0.25	0.23	0.23	0.22	0.21	0.22
Stable baseli	0.23	0.25	0.21	0.23	0.24	0.22	0.21	0.23
0	0.7	0.8	0.7	0.8	0.7	0.8	0.7	0.7
60	0.6	1	0.5	1	0.7	0.9	0.7	1
120	0.5	1	0.4	0.7	0.65	0.7	0.55	0.9
180	0.5	0.8	0.35	0.5	0.6	0.45	0.5	0.9
240	0.4	0.8	0.45	0.25	0.65	0.5	0.5	0.94
PaO2 (mmHg)								
Post-surgery			80	60				

Stable baseli	56	68	64	59	64	56	56	79
0	101	69	58	66	59	71	53	94
60	124	92	60	95	83	108	91	92
120	na (machine)	106	49	69	83	87	68	64
180	161	na		82	66	60	64	63
240	136	na	60	58	57	64	68	72

PaCO2 (mmHg)

Post-surgery			32.6	37.1				
Stable baseli	47.6	43.7	39.9	44.7	37.8	32.1	34.4	39.9
0	45.3	59.4	41.8	47.8	37.8	34.8	35	32.4
60	44.3	72.3	34.3	47.7	45.2	49	42.9	51.6
120		57.1	40.2	44.2	50.5	36.3	43.8	51.6
180	36.7	na		42.8	52.7	37.8	44.8	50.6
240	37.3	na	39.7	41.9	49.1	43.3	44	49.5

OI

Post-surgery			2.8125	3.565				
Stable baseli	3.98392857	3.78676471	2.8875	3.66440678	3.675	4.00714286	3.6	2.76582278
0	11.7821782	16.9275362	16.41379	15.7575758	20.5254237	19.4929577	19.8113208	11.1702128
60	6.77419355	21.7391304	10.83333	15.4736842	10.9638554	13.4166667	9.92307692	18.9130435
120		18.8679245	9.795918	11.6666667	9.08433735	9.65517241	9.30147059	24.1875
180	3.72670808	na		7.07317073	10.9090909	9	8.984375	23
240	3.52941177	na	8.7	5.17241379	13.5701754	9.375	8.52941176	20.2361111

AaDO2 (mmHg)

Post-surgery			57.5	57.615				
Stable baseli	48.49	55.625	35.855	49.115	59.87	60.735	50.73	35.115
5min post	349	431	395	381	386	373	388	387
0	341.475	427.15	388.85	444.65	392.85	455.9	402.35	364.6
60	248.425	530.625	253.625	558.375	359.6	472.45	354.475	556.5
120		535.625	185.95	374.85	317.325	366.725	269.4	513.2
180	149.625	na	249.55	221	295.925	213.6	236.5	515.45
240	102.575	na	211.225	67.875	345.075	238.375	233.5	536.345

pH

Post-surgery			7.538	7.49				
Stable baseli	7.31	7.31	7.426	7.38	7.455	7.406	7.503	7.422
0	7.32	7.13	7.419	7.3	7.409	7.342	7.427	7.418
60	7.32	7.11	7.379	7.3	7.335	7.216	7.37	7.191
120		7.17	7.278	7.31	7.327	7.3	7.376	7.144
180	7.37	na		7.3	7.315	7.283	7.377	7.107
240	7.39	na	7.249	7.33	7.347	7.239	7.374	7.06

HCO3

Post-surgery			27.7	28.4				
Stable baseli	24	21.8	26.2	26.4	26.5	20.2	27	26
0	23.4		27.1	23.5	23.9	18.9	23.1	20.9
60	22.8	22.7	20.2	23.6	24.1	19.9	24.8	19.7
120		20.9	18.8	22.1	26.4	17.8	25.6	17.7
180	22.6			21.1	26.8	17.9	26.3	16
240	22.3		17.3	22.1	26.9	18.5	25.7	14

BE

Post-surgery			5	5				
Stable baseli	-2	-5	2	1	3	-5	4	2
0	-3		3	-3	-1	-7	-1	-4
60	-3	-7	-5	-3	-2	-8	0	-8
120		-8	-8	-4	0	-9	0	-11
180	-3		-10	-5	1	-9	1	-14
240	-3			-4	1	-9	0	-16

Hemoglobin (g/L)

Post-surgery		82	91	95	61	67	78	66
Stable baseli	96	93	77	96	68	67	81	69
0	106	88	78	96	65	69	82	73
60	101	87	83	101	68	75	94	88
120	99	82	82	101	59	75	94	80
180	91	76	81	92	54	70	89	71
240	85	75	78	92	49	66	86	72

VT (ml/kg)

Post-surgery	12.7058824	14.5789474	13.76471	13.8823529	14	13.4736842	12.8235294	12.5263158
Stable baseli	12.6470588	14.5789474	13.88235	13.8823529	16.1764706	13.5263158	15.0588235	14.3684211
0	3.35294118	3.78947368	3.941176	3.17647059	3.76470588	3.15789474	3.88235294	2.78947368
60	3.82352941	4.10526316	4.235294	4.11764706	4.52941176	4	4.47058824	3.78947368
120	3.82352941	4.36842105	3.823529	4.235294118	4.35294118	4.31578947	4.47058824	3.68421053
180	3.88235294	4.42105263	3.882353	4.23529412	4.29411765	4.31578947	4.47058824	3.68421053
240	3.88235294	4.68421053	3.941176	4.17647059	4.35294118	4.05263158	4.58823529	3.68421053

MV (ml/kg/min)

Post-surgery								
Stable baseli	695.588235	874.736842	555.2941	694.117647	889.705882	973.894737	903.529412	862.105263
0	2011.76471	2273.68421	2364.706	1905.88235	2258.82353	1894.73684	2329.41176	1673.68421
60	2294.11765	2463.15789	2541.176	2470.58824	2717.64706	2400	2682.35294	2273.68421
120	2294.11765	2621.05263	2294.118	2541.17647	2611.76471	2589.47368	2682.35294	2210.52632
180	2329.41176	2652.63158	2329.412	2541.17647	2576.47059	2589.47368	2682.35294	2210.52632

240	2329.41176	2810.52632	2364.706	2505.88235	2611.76471	2431.57895	2752.94118	2210.52632

DCO2 (ml^2/sec)

0	324	532	448	280	396	360	435	336
60	422	608	518	490	592	577	577	518
120	435	672	422	532	547	672	562	490
180	435	688	435	578	532	672	577	490
240	448	739	448	504	532	592	608	504

Amplitude (cmH2O)

0	30	55	50	30	40	40	40	40
60	30	60	45	45	51	55	50	45
120	29	70	40	46	51	55	50	46
180	30	68	41	46	50	56	51	46
240	30	66	41	46	50	50	51	46

Temp ©						38.3		
Post-surgery	39.4	40.2	38.3	38.4	39.8	39.8	37.9	38.7
Stable baseli	40	40.3	38.2	39.8	39.5	39.3	40.1	40.8
0	39.3	39.5	38.7	39.1	38.7	39.1	39.3	38.3
60	38.1	39.1	39.4	39.2	39.6	39.1	39.9	40.1
120	40.2	38.9	38.6	41.1	39.2	38.9	39.6	39.7
180	40	38.5	39.4	39	38.9	39.2	39.7	38.7
240	39.4	39.8	40.7	38.1	39	39.5	38.6	39.6

MILLAR

Tau (ms)

Stable baseli	15.74	15.51	23.43	17.23	17.43	23.73	15.32	13.87
0	16.56	17.92	56.81	20.52	23.97	22.27	24.83	19.62
60	14.48	17	33.15	17.97	17.29	18.22	14.79	13.41
120	14.01	21.34	8.131	15.43	16.07	18.99	15.86	15.57
180	13.89	20.51	23.55	19.98	2703	17.37	16.35	17.58
240	16.92	18.81	20.1	22.18	15.44	15.8		16.91

Stroke volume index (ml/kg)

Stable baseli	0.40823529	0.39715789	0.503941	0.41111765	0.38670588	0.54052632	0.473	0.44247368
0	0.27147059	0.49278947	0.371353	0.34647059	0.24523529	0.42442105	0.43047059	0.30894737
60	0.23570588	0.56368421	0.443647	0.327	0.27658824	0.31352632	0.42523529	0.25468421
120	0.20882353	0.53894737	0.317941	0.44017647	0.287	0.35389474	0.31917647	0.26231579
180	0.34376471	0.54052632	0.278471	0.53164706	0.47735294	0.39015789	0.36347059	0.29863158
240	0.54247059	0.57105263	0.312176	0.47805882	0.48788235	0.405		0.29447368

Stroke work (mmHg*ml)

Stable baseli	42.48	57.15	48.15	31.35	36.28	55.79	52.22	52.61
0	29.83	55.69	29.38	29.69	18.32	45.4	35.69	31.33
60	13.31	71.47	33.52	31.06	15.54	32.52	54.66	26.33
120	14.46	54.17	23.71	38.08	20.44	33.91	29.89	22.7
180	35.75	56.99	22.27	43.54	47.19	39.4	31.44	22.56
240	60.92	67.71	18.69	40.83	42.89	35.32		22.76

MILLAR DATA

Stroke work (% change from baseline)

Stable baseli	1	1	1	1	1	1	1	1
0	0.70023529	0.97535396	0.611215	0.94765401	0.49741356	0.81329511	0.68332376	0.59364898
60	0.31364706	1.2499563	0.695327	0.99968082	0.42281514	0.58018276	1.04652499	0.50028523
120	0.34070588	0.94546408	0.49242	1.20906479	0.55649333	0.60186347	0.57227647	0.4331622
180	0.84117647	0.99563013	0.461682	1.38653048	1.28233052	0.70614585	0.6019529	0.42802814
240	1.27058824	1.17636777	0.388162	1.24736674	1.16988837	0.6310697	1.01608271	0.4327819

Stroke volume (% change from baseline)

Stable baseli	1	1	1	1	1	1	1	1
0	0.66450497	1.24069413	0.737623	0.8434907	0.62884731	0.78349515	0.91008581	0.69346973
60	0.57861363	1.41873096	0.880546	0.79055794	0.70911285	0.57456311	0.89876881	0.57535387
120	0.51131287	1.35647106	0.63113	1.05965665	0.73611949	0.65446602	0.67454297	0.59343404
180	0.84248451	1.35912041	0.552546	1.28726753	1.22314424	0.72058252	0.76843676	0.67467587
240	1.18994091	1.40813353	0.619804	1.17496423	1.25196138	0.7468932	1.07412013	0.66563578

End-diastolic volume (% change from baseline)

Stable baseli	1	1	1	1	1	1	1	1
0	0.91861436	0.9478299	0.899511	0.99158111	0.89861647	0.85728796	0.95216708	0.85028902
60	0.94240401	0.95024112	0.941789	1.02443532	0.89521433	0.78237684	0.89737256	0.75751445
120	0.97704508	0.95703639	0.878054	1.04271047	0.9278748	0.86846115	0.87963171	0.74942197
180	1.04966611	0.98662867	0.841513	1.06570842	1.01655704	0.86008126	0.8852459	0.76589595
240	1.07929883	0.98838229	0.901636	1.02094456	1.02880472	0.86693753	0.85066247	0.77630058

End-diastolic pressure (% change from baseline)

Stable baseli	1	1	1	1	1	1	1	1
0	1.26618123	0.79870464	1.116706	1.21841637	1.08721435	1.09922601	0.87636553	0.88409284

60	1.41213592	0.77896577	1.025237	1.26645907	0.78897888	0.77414861	0.59909832	0.66855364
120	1.23996764	0.94397039	0.950355	1.12477758	0.65866358	0.77399381	0.61990636	0.76804416
180	1.47216828	1.00318701	0.77346	1.37344306	1.11426092	0.77693498	0.636206	0.79705633
240	1.5315534	0.940989	0.833531	1.20774021	1.02458779	0.59133127	0.76833709	0.81135013

Ejection fraction (% change from baseline)

Stable baseli	1	1	1	1	1	1	1	1
0	0.7286115	1.26235294	0.825318	0.85227273	0.67313916	0.86160211	0.9688196	0.79493566
60	0.63008415	1.44529412	0.934771	0.78267045	0.77605178	0.68446785	1.02004454	0.73100872
120	0.53625526	1.37352941	0.73853	1.03551136	0.77540453	0.75253855	0.77004454	0.76670818
180	0.82117812	1.33705882	0.630735	1.23224432	1.20194175	0.81722452	0.86971047	0.85097551
240	1.10308555	1.37941176	0.67717	1.17045455	1.22330097	0.85520873	1.22605791	0.83478622

Max dP/dt (% change from baseline)

Stable baseli	1	1	1	1	1	1	1	1
0	0.98580786	0.742763	0.840965	0.7531337	0.91789993	0.8945885	0.60291121	0.74096079
60	1.05376638	0.8150153	0.733911	0.35584958	1.2769556	0.87232244	1.11528384	0.84756408
120	1.0253821	0.43892681	0.784963	0.33025766	1.36997886	0.54847802	0.7874818	0.49125785
180	0.84688865	0.44457519	0.914604	0.27872563	1.27343199	0.66685457	0.69199418	0.38618231
240	0.8018559	0.61896917	0.425743	0.30954039	1.10817477	0.8427283	0.84279476	0.37752504

Min dP/dt (% change from baseline)

Stable baseli	1	1	1	1	1	1	1	1
0	1.32159443	0.4635751	0.852703	0.85865724	0.65579904	0.78754081	0.70790698	0.76253727
60	1.50425697	0.45972401	0.929486	1.02756184	0.61589168	0.77339499	1.24906977	1.01789103
120	1.56559598	0.34579589	0.950379	0.99929329	0.609478	0.77040261	0.83418605	0.69991868
180	1.22387771	0.33632863	1.585531	0.70106007	0.85640477	0.79760609	0.59953488	0.54648956
240	0.49690402	0.375	0.644816	0.53144876	0.74701586	0.68199129	1.04325581	0.56004337

Tau (% change from baseline)

Stable baseli	1	1	1	1	1	1	1	1
0	1.05212969	1.15132003	2.420068	1.19094602	1.37751004	0.93563315	1.62181581	1.42507205
60	0.92053401	1.09401159	1.385629	1.04759141	0.99196787	0.769878	0.96603527	0.96541787
120	0.8906548	1.37540245	0.344005	0.89901335	0.92139989	0.80227177	1.03592423	1.12247839
180	0.88239034	1.32066967	1.002126	1.16076611	144.520941	0.73033235	1.06727629	1.26729107
240	1.14367451	1.20412106	0.855442	1.28612885	0.88582903	0.66344131	1.02808622	1.21685879
