# Supplementary material for: Hemodynamic effects of high frequency oscillatory ventilation with volume guarantee in a piglet model of respiratory distress syndrome
Source: PLoS One. 2021 Feb 16;16(2):e0246996. doi: 10.1371/journal.pone.0246996 (PMC7886162; doi:10.1371/journal.pone.0246996)
Supplement: S2 Table — (PDF) [file pone.0246996.s003.pdf]

Wt (kg)	1.8	2	1.9	2	1.8	1.6	2.1	2
Age (days)	2	3	1	3	2	1	2	1
Gender (1=M,	1	2	1	1	1	1	1	1
Treatment (1	HFOV+VG	HFOV+VG	HFOV+VG	HFOV+VG	HFOV+VG	HFOV+VG	HFOV+VG	HFOV+VG
Surgical time (	100	80	85	65	80	70	65	55
Lavage time (r	68	60	100	60	55	60	45	40
Lavage numb	10	11	23	10	12	16	10	8
HR (bpm)								
Post-surgery	191	216	186	276	250	218	204	171
Stable baseli	244	249	217	240	263	202	204	191
0	220	243	218	265	226	222	217	192
60	235	213	246	273	236	217	175	182
120	238	229	249	231	246	233	182	238
180	244	215	248	217	216	261	211	248
240	233	250		158	180	263	231	191
Mean bp (mmHg)								
Post-surgery	73	87	46	71	54	60	64	49
Stable baseli	73	83	56	72	59	52	64	59
0	58	73	56	75	60	54	55	60
60	37	73	50	70	52	55	58	54
120	37	48	56	32	55	44	57	60
180	47	34	43	23	34	48	56	46
240	47	32		15	29	44	49	32
Systolic bp (mmHg)								
Post-surgery	98	110	67	92	73	79	79	65
Stable baseli	82	109	79	99	74	73	95	84
0	70	98	75	101	85	72	80	79
60	51	97	64	99	73	74	86	69
120	61	73	60	49	82	63	83	76
180	53	61	46	37	58	67	83	65
240	61	52		23	52	64	77	49
Diastolic bp (mmHg)								
Post-surgery	52	69	33	58	44	45	53	38
Stable baseli	65	69	42	56	51	38	42	44
0	49	60	43	61	49	42	37	47
60	27	58	41	54	42	42	37	45
120	32	34	53	23	42	32	38	49
180	42	22	42	15	21	34	36	38
240	36	19		11	18	31	30	24
CO (ml/kg/min)								
Stable baseli	46.5111111	114.5	102.105263	79	115.5556	113.125	70.952381	124.5

0	24.4388889	49.5	66.3157895	96	80	115	86.1904762	100.5
60	28.6222222	48	86.8421053	108	96.11111	136.25	70.4761905	74.5
120	34.1555556	37	92.6315789	89	93.33333	190	97.1428571	90
180	37.6666667	35	139.473684	29	70.55556	198.75	112.857143	137.5
240	37.8444444	55.5		18.5	57.22222	201.875	115.714286	126.5

NIRS - cerebral (% saturation)

Post-surgery	55	40	38	38	28	34	42	39
Stable baseli	54	47	44	35	46	35	38	38
0	44	36	40	35	38	38	40	43
60	45	51	42	35	41	33	43	42
120	45	52	43	28	35	33	35	38
180	45	46	34	21	40	45	37	29
240	21	36		16	28	43	42	24

CA flow (ml/kg/min)

Post-surgery	37.2222222	60.5	32.6315789	34.5		36.875	51.4285714	28.5
Stable baseli	35	47	33.6842105	40.5	27.77778	42.5	44.2857143	29
0	12.7777778	48.5	25.7894737	42	37.22222	28.75	28.5714286	27.5
60	9.44444444	54.5	15.2631579	na	30	35.625	20.4761905	13.5
120	8.888888889	na	24.7368421	5.5	25.55556	41.25	20.952381	12.5
180	10.5555556	na	0.52631579	0.5	7.777778	25	19.5238095	9.5
240	27.7777778	na		1	11.66667	21.875	18.5714286	5

Lactate (mmol/L)

Post-surgery	3.3							
Stable baseline		3.88	4.32	4.11	9.11	5.1	5.36	4.09
0	4	5.21	4.79	7.13	8.08	4.42	4.92	4.06
60	3.51	9.56	2.66	8.94	7.74	5.4	4.68	3.57
120		9.42	2.35	10.3	6.51	na	3.88	3.95
180		na	na	na	6.15	3.9	4.21	na
240	6.3	10.89	na	17.1	7.62	3.09	2.92	5.14

SaO2 (%)

Post-surgery	99.5				94.2		98.5	97.5
Stable baseline		97.5	98.3	91.2	97.7	96.5	88.7	91
0	97.1	79.6	94.7	95.8	92.2	98.2	98.5	95.1
60	98	97.2	94.3	95	97.3	98.7	100	100
120	96	96.1	93.2	96.2	95.7	100	96.2	98.3
180	93	96	92.6	100	99.1	98.8	88.7	91.6
240	98	83.8		84.8	95.4	100	92.1	95.7

Paw (cmH2O)

Post-surgery	9.3	8.9	8.6	9.3	9.4	8.7	8.9	9.4
Stable baseli	9.9	9.4	9	10	9.5	9.1	9.3	10.8
0	19	18	16.3	15.7	13.5	13.2	14.2	14.6
60	19	20	13	15	16	12	14.8	15.2
120	19	19	13	14	13	12.5	11.8	12
180	19	18	14	12	12	12.5	11.8	12
240	19	13		11.5	12	12	11.9	11.4
PEEP (cm H2O)								
Post-surgery	5.9	5.6	5.6	5.4	5.7	5.6	5.5	5.6
Stable baseli	5.6	5.5	5.7	5.4	5.4	5.6	5.4	5.5
0	na	hfo	hfo	hfo	hfo	hfo	hfo	hfo
60	na							
120	na							
180	na							
240	na							
PIP (cm H2O)								
Post-surgery	na	20	18	23	22	18	20	23
Stable baseli	na	22	20	27	20	19	22	26
0	na	hfo	hfo	hfo	hfo	hfo	hfo	hfo
60	na							
120	na							
180	na							
240	na							
FiO2								
Post-surgery	0.31	0.23	0.23	0.21	0.23	0.23	0.21	0.21
Stable baseli	0.31	0.32	0.25	0.25	0.23	0.23	0.21	0.21
0	0.8	1	0.8	0.7	0.7	0.7	0.7	0.7
60	0.77	0.9	0.9	0.7	0.9	0.6	1	0.9
120	0.7	0.9	0.9	0.65	0.8	0.96	0.6	0.4
180	0.65	0.9	0.9	0.65	0.85	0.75	0.4	0.3
240	0.65	0.8		0.6	0.85	0.65	0.45	0.3
PaO2 (mmHg)								
Post-surgery	89							
Stable baseline		84	76	61	75	62	47	52
0	55	56	62	77	64	70	75	73
60	87	115	65	78	83	77	134	242
120		108	53	84	73	50	74	85
180			na		86	76	56	na
240	139	74		73	70	83	59	68

PaCO2 (mmHg)

Post-surgery	59.4							
Stable baseline		49.3	42.1	42.2	39.8	46.9	36.6	35
0	41.8	53.2	34.8	39.8	43.3	50	42.8	40.4
60	53.6	65.3	40.2	61.2	51.6	45.5	58.9	39.4
120		60.2	43.7	51.2	49.1	47.6	50.3	35.1
180			na		48.3	43.4	47.2	na
240	49.6	65.3		38.8	44.4	42	52.8	30.5

OI

Post-surgery	3.23932584							
Stable baseline		3.58095238	2.96052632	4.09836066	2.913333	3.37580645	4.15531915	4.36153846
0	27.6363636	32.1428571	21.0322581	14.2727273	14.76563	13.2	13.2533333	14
60	16.816092	15.6521739	18	13.4615385	17.3494	9.35064935	11.0447761	5.65289256
120		15.8333333	22.0754717	10.8333333	14.24658	24	9.56756757	5.64705882
180		na			11.86047	12.3355263	8.42857143	
240	8.88489209	14.0540541		9.45205479	14.57143	9.39759036	9.07627119	5.02941176

AaDO2 (mmHg)

Post-surgery	57.78							
Stable baseline		82.535	49.625	64.5	39.24	43.365	56.98	53.98
5min post	455	446	467	378	383	362	361	366
0	463.15	590.5	464.9	372.35	380.975	366.6	370.6	375.6
60	395.01	445.075	526.45	344.6	494.2	293.925	505.375	350.45
120		458.45	534.075	315.45	436.025	574.98	290.925	156.325
180		na			459.675	404.5	170.2	
240	262.45	414.775		306.3	480.55	327.95	195.85	107.775

pH

Post-surgery	7.37							
Stable baseline		7.3	7.41	7.36	7.32	7.394	7.499	7.436
0	7.33	7.19	7.37	7.26	7.253	7.317	7.384	7.326
60	7.24	6.99	7.34	7.09	7.191	7.358	7.239	7.314
120		7.03	7.31	7.11	7.24	7.317	7.319	7.352
180		na			7.254	7.37	7.324	
240	7.22	6.96		6.93	7.244	7.39	7.327	7.336

HCO3

Post-surgery	34.4							
Stable baseline		24.2	26.9	23.9	20.5	28.7	28.5	23.6
0	21.8	20.4	20.2	18	19.1	25.5	25.5	21.1
60	22.9	15.9	21.9	18.4	19.8	25.6	25.2	20
120		16.2	22.2	16.4	21.1	24.4	25.8	19.4

180					21.4	25.2	24.5	
240	20.4	14.8		8.1	19.2	25.7	27.7	16.3
BE								
Post-surgery	9							
Stable baseline		-2	2	-2	-6	4	5	-1
0	-4	-8	-5	-9	-8	-1	0	-5
60	-5	-16	-4	-11	-8	0	-2	-6
120		-14	-4	-13	-6	-2	0	-6
180					-6	0	-1	
240	-7	-17		-24	-8	1	2	-10
Hemoglobin (g/L)								
Post-surgery	85				66		75	82
Stable baseline		68	84	81	59	63	75	87
0	95	66	82	67	59	62	76	89
60	90	62	92	65	56	64	77	98
120		60	88	60	54	67	80	96
180		59	77	55	48	65	80	94
240	81	55		52	46	62	81	84
VT (ml/kg)								
Post-surgery	14.1666667	12.6	12.4736842	13.8	14.27778	12.4375	13.1904762	12.75
Stable baseli	14.1666667	13.75	13.4736842	13.6	15.22222	12.5	13.0952381	12.8
0	2.94444444	3.95	4.47368421	4	3	4	3.14285714	3
60	4.88888889	4.3	4.47368421	4	4	3.9375	3.66666667	3.95
120	4.77777778	4.15	4.47368421	4.25	4	4	3.71428571	4
180	4.72222222	4.2	4.36842105	4.3	4	4	3.80952381	3.95
240	4.777777778	4.25		4.5	4	4	3.76190476	3.95
MV (ml/kg/min)								
Post-surgery								
Stable baseli	1275	687.5	673.684211	748	913.3333	687.5	720.238095	844.8
0	1766.666667	2370	2684.21053	2400	1800	2400	1885.71429	1800
60	2933.333333	2580	2684.21053	2400	2400	2362.5	2200	2370
120	2866.66667	2490	2684.21053	2550	2400	2400	2228.57143	2400
180	2833.33333	2520	2621.05263	2580	2400	2400	2285.71429	2370
240	2866.66667	2550		2700	2400	2400	2257.14286	2370
DCO2 (ml^2/sec)								
0	290	532	739	624	291	396	384	396
60	774	739	722	624	518	396	592	640
120	739	688	688	705	518	396	608	640
180	722	755	672	756	518	396	624	640
240	739	722		810	518	396	624	562

Amplitude (cmH2O)

0	na	na	73	80	34	50	66	55
60	na	na	74	61	50	80	76	71
120	na	na	73	73	49	80	74	61
180	na	na	73	71	51	94	68	68
240	na	na	na	66	49	94	69	75

Temp ©

Post-surgery	38.9	38.7	39	39.7	39.7	38.8	37.6	37.3
Stable baseli	40.6	40.2	39.7	40.4	39.3	38.5	40.9	39.7
0	38.2	39.4	39.9	38.4	39	38	39.2	39.2
60	38.1	38.5	39.6	40.4	39.3	39.1	39.7	39
120	38.1	39.7	39.4	40.1	39	38.4	39	39.4
180	38.3	41	39.3	38.6	38.6	38.6	39	40.2
240	38.4	40.2		38.1	39.1	38.6	39.5	38

MILLAR

Tau (ms)

Stable baseli	14.81	13.06	17.69	14.67	19.12	19.48	16.59	14.79
0	23.72	16.3	26.23	14.39	20.24	21.26	19.17	27.5
60	22.46	17.22	17.16	10.31	16.34	13.99	41	20.24
120	19.75	18.75	16.04	16.11	16.95	16.29	22.8	17.24
180	18.03	16.79	18.31	23.17	14.94	17.39	22.84	11.43
240	17.19	14.9		32.74	14.24	16.12	17.97	18.2

Stroke volume index (ml/kg)

Stable baseli	0.03496667	0.4121	0.44715789	0.35365	0.298611	0.4068125	0.424	0.69
0	0.02061111	0.31045	0.30689474	0.36385	0.343944	0.4945	0.42166667	0.5165
60	0.02412778	0.23235	0.34089474	0.3657	0.346611	0.53475	0.23285714	0.40795
120	0.02804444	0.13725	0.38157895	0.33875	0.370222	0.64875	0.29361905	0.35695
180	0.03082222	0.13805	0.46778947	0.3464	0.320333	0.758125	0.28042857	0.5185
240	0.03232778	0.30235	0	0.1512	0.325889	0.751875	0.33871429	0.5845

Stroke work (mmHg*ml)

Stable baseli	3.745	43.7	39.37	43.7	28.71	34.08	50.23	78.8
0	1.206	37.36	26.7	51.14	30.09	42.13	47.68	57.07
60	1.439	30.14	23.86	46.79	29.71	50.68	18	38.52
120	2.005	7.041	33.84	24.34	32.04	57.45	27.33	38.92
180	2.348	9.48	34.03	19.63	25.58	64.44	16.41	46.34
240	2.748	25.26		5.655	25.08	65.4	30.16	43.18

MILLAR DATA

Stroke work (% change from baseline)

Stable baseli	1	1	1	1	1	1	1	1
0	0.32475936	0.85023934	0.67597765	1.15080092	0.94845	0.99505067	0.94553073	0.72898146
60	0.38508021	0.68566218	0.60639919	1.07070938	0.939911	1.20009427	0.35654429	0.49199898
120	0.53582888	0.15947116	0.85931945	0.55675057	1.013283	1.35446618	0.53810854	0.49339599
180	0.62780749	0.2178026	0.86541392	0.44897025	0.809614	1.5180297	0.32721468	0.58763018
240	0.73850267	0.40893549		0.12881007	0.777356	1.54018383	0.60215483	0.5511811

Stroke volume (% change from baseline)

Stable baseli	1	1	1	1	1	1	1	1
0	0.59090909	0.75230471	0.68259948	1.01696593	1.051034	0.97531936	1.00483635	0.75144928
60	0.69071837	0.56344008	0.76206734	1.03407324	1.057986	1.06089545	0.54785738	0.59355072
120	0.80228862	0.33224163	0.85377914	0.95786795	1.141573	1.28860226	0.69103588	0.51550725
180	0.88111888	0.33575934	1.04591476	0.97907536	0.977789	1.50440283	0.66381734	0.75144928
240	0.92498411	0.51346434		0.42584476	0.984401	1.4920005	0.80159712	0.84855072

End-diastolic volume (% change from baseline)

Stable baseli	1	1	1	1	1	1	1	1
0	0.95452196	0.96784777	1.02609236	0.93541203	1.031297	1.0201594	1.00503145	0.87245971
60	0.95891473	0.96948819	0.92462207	0.98094531	1.04183	1.01312705	0.9672956	0.77084793
120	0.9630491	0.90485564	0.94160282	0.9816877	1.050858	1.06540084	0.84549266	0.80798879
180	0.9630491	0.91502625	0.95816939	0.99356595	1.109239	1.04571027	0.81257862	0.78416258
240	0.96408269	0.875		1.0081663	1.17093	1.0503985	0.85220126	0.82177061

End-diastolic pressure (% change from baseline)

Stable baseli	1	1	1	1	1	1	1	1
0	1.44090909	1.13193735	1.17675107	1.21385154	1.17514	0.9587868	1.22400135	1.11181602
60	1.26072727	1.45658836	0.84824071	1.07570455	1.200271	0.9102587	3.5530086	0.83267248
120	1.31490909	1.38729997	0.83360737	1.0456806	1.091436	0.9396967	1.38344851	0.70103093
180	1.38690909	1.28021791	1.02088129	1.18548536	1.280108	0.98394291	3.01028148	0.60111023
240	1.51545455	1.24940415		1.18401179	1.367872	1.00035682	1.3153548	0.79962992

Ejection fraction (% change from baseline)

Stable baseli	1	1	1	1	1	1	1	1
0	0.88963964	0.94498256	0.97062873	1.13252508	0.890796	1.11992168	1.07733463	0.97733127
60	0.89001502	0.82177451	1.12161542	1.13545151	0.927457	1.24473813	1.73735409	0.94538897
120	0.91403904	0.89655172	1.1353832	0.97366221	0.951248	1.24180127	1.03793774	1.15043792
180	0.91779279	0.85625726	1.15098669	0.91973244	0.842434	1.28193833	1.91001946	1.26790314

240	0.87837838	0.96358001		0.73745819	0.691888	1.27998042	1.12986381	0.96239052

Max dP/dt (% change from baseline)

Stable baseli	1	1	1	1	1	1	1	1
0	1.053374	1.40935006	1.46327841	1.41644794	0.581049	1.27192982	0.99693292	1.26566674
60	0.86313382	1.56841505	1.29949735	1.13560805	0.583935	1.45906433	2.33364449	1.60673926
120	0.85169653	2.44811859	1.2051103	0.66342957	0.533911	1.13060429	1.47753034	2.01047873
180	0.7945101	2.14994299	1.26221726	0.50831146	0.506975	0.98635478	1.37484998	1.32484076
240	0.88982082	0.93443558		0.86509186	0.455123	0.89668616	1.24443259	0.89932196

Min dP/dt (% change from baseline)

Stable baseli	1	1	1	1	1	1	1	1
0	0.39677493	0.88881564	1.35333613	1.1876148	0.692131	1.23824593	0.63920241	0.8041897
60	0.35339541	0.67391782	1.07217793	0.92337969	0.699598	1.69122966	1.0662152	0.62990981
120	0.410402	0.604922	1.07847251	0.4069798	0.626364	1.47016275	1.07317532	0.96654059
180	0.43811038	0.56910569	0.99958036	0.24746786	0.539058	1.41772152	1.96576373	0.58539424
240	0.50079491	0.45638321		0.143217	0.431648	1.40596745	2.14446953	0.39453011

Tau (% change from baseline)

Stable baseli	1	1	1	1	1	1	1	1
0	1.59649123	1.24980843	1.48925339	0.87866394	1.05312	1.08841463	1.15167866	1.80797836
60	1.51956815	1.3210728	0.97002262	0.70279482	0.842702	0.71138211	2.55635492	1.36646383
120	1.33333333	1.43524904	0.9061086	1.09679618	0.870036	0.8277439	1.43045564	1.16497634
180	1.2145749	1.28122605	1.03167421	1.58486708	0.769985	0.88414634	1.3705036	0.77146721
240	1.16126856	1.0789272		2.23040218	0.732336	0.81910569	1.07553957	1.22853279
