# Supplementary material for: Hemodynamic effects of high frequency oscillatory ventilation with volume guarantee in a piglet model of respiratory distress syndrome
Source: PLoS One. 2021 Feb 16;16(2):e0246996. doi: 10.1371/journal.pone.0246996 (PMC7886162; doi:10.1371/journal.pone.0246996)
Supplement: S3 Table — (PDF) [file pone.0246996.s004.pdf]

Wt (kg)	1.5	1.9	1.9	1.8	1.7	2.2
Age (days)	1	2	2	2	2	3
Gender (1=M, 2=F)		2	2	1	1	1
Treatment	SHAM	SHAM	SHAM	SHAM	SHAM	SHAM
Surgical tim	135	65	70	80	65	55
Lavage time	na	na	na	na	na	na
Lavage nu	na	na	na	na	na	na
HR (bpm)						
Post-surge	221	250	200	197	234	192
Stable bas	208	238	216	203	193	210
0	212	237	244	168	224	162
60	207	236	261	183	204	187
120	180	274	253	191	219	210
180	218	214	248	219	231	220
240	240	197	256	250	203	268
Mean bp (mmHg)						
Post-surge	72	83	74	69	64	103
Stable bas	63	67	58	70	66	69
0	63	70	57	59	64	66
60	56	52	44	55	48	73
120	46	51	44	50	54	48
180	46	34	36	52	61	45
240	40	33	39	51	44	49
Systolic bp (mmHg)						
Post-surge	94	109	101	94	69	86
Stable bas	89	92	84	91	89	95
0	85	96	75	83	86	89
60	79	74	61	76	53	93
120	67	76	66	79	79	66
180	71	55	39	83	84	65
240	51	58	58	81	79	67
Diastolic bp (mmHg)						
Post-surge	55	65	56	30	60	47
Stable bas	48	50	43	55	48	49
0	52	54	46	43	50	53

60	41	37	34	40	44	59
120	31	35	33	33	37	36
180	31	21	33	34	42	32
240	32	20	27	32	28	33

CO (ml/kg/min)

Stable bas	141.7778	90	92.63158	60.55556	51.17647	55.9090909
0	152	75.78947	76.84211	78.33333	78.23529	29.5454545
60	149.7778	83.68421	82.10526	74.44444	71.17647	45
120	139.1111	93.68421	75.78947	73.88889	92.35294	85.4545455
180	152.4444	67.89474	67.36842	101.1111	111.7647	108.636364
240	186.6667	76.84211	86.31579	107.7778	92.94118	106.818182

NIRS - cerebral (% saturation)

Post-surge	54	44	44	39	40	45
Stable bas	47	40	38	40	40	45
0	47	35	36	38	39	39
60	49	38	36	36	45	39
120	51	36	35	41	49	40
180	48	26	28	41	50	44
240	28	22	24	47	37	47

CA flow (ml/kg/min)

Post-surge	36	43.68421	35.78947	38.88889	40.58824	36.8181818
Stable bas	20.66667	39.47368	33.15789	42.77778	41.17647	35.4545455
0	20	28.94737	26.31579	22.22222	38.23529	26.3636364
60	22	16.84211	27.89474	26.66667	38.23529	19.0909091
120	19.33333	24.73684	18.42105	16.66667	43.52941	27.7272727
180	22	4.736842	17.89474	35	55.29412	21.8181818
240	18.66667	7.368421	15.78947	22.77778	40.58824	25

Lactate (mmol/L)

Post-surge	2					
Stable bas	2.9	4.09	3.82	7.43	6.49	3.31
0	2.88	4.63	4.58		5.55	2.91
60	1.99	4.1	4.45	9.96	4.17	2.87
120		5.04	4.22			
180		3.26	3.3			

240	1.68		4.48	9.54	1.94	1.8

SaO2 (%)						
Post-surge	98.1	94	94	90.9	94.2	92.2
Stable bas	97.2	96.2	94	93.6	93.5	89
0	95	91.7	89	94.6	97.5	90.8
60	94.4	96	91	90.4	97.4	98.4
120	94.6	84	83	93.5	95.5	96.5
180	91	93	92	92.8	97.5	96
240	87.6	95	94	95	87.8	97.1

Paw (cmH2O)						
Post-surge	8.4	8.9	9.4	9.8	9.9	9.2
Stable bas	8.6	9.5	9.5	10.4	10.1	9
0	8.8	9.7	9.6	10.3	11.6	9.2
60	8.7	8.7	9.8	10	10.7	8.9
120	8.7	8.9	10.1	11	10.9	11.3
180	8.7	8.8	9.3	12.2	10.5	9.1
240	8.6	8.9	9.7	10.3	10.6	12.5

PEEP (cm H2O)						
Post-surge	6	5.6	5.6	5.5	5.5	5.5
Stable bas	6	5.6	5.5	5.5	5.5	5.4
0	6	5.5	5.6	5.4	5.4	5.4
60	6	5.5	5.4	5.6	5.5	5.4
120	6	5.5	5.5	5.7	5.6	5.6
180	6	5.5	5.4	6.6	5.6	5.5
240	6	5.5	5.6	6.4	5.6	5.5

PIP (cm H2O)						
Post-surge	17	22	24	22	23	19
Stable bas	17	26	25	24	23	19
0	19	27	26	24	31	20
60	19	18	26	22	25	18
120	20	18	26	21	26	24
180	19	18	22	23	24	19
240	18	18	24	21	25	35

FiO2

Post-surge	0.25	0.23	0.23	0.22	0.23	0.23
Stable bas	0.25	0.23	0.23	0.22	0.23	0.23
0	0.25	0.23	0.25	0.23	0.23	0.24
60	0.25	0.23	0.25	0.23	0.23	0.24
120	0.25	0.25	0.25	0.25	0.23	0.24
180	0.25	0.25	0.25	0.25	0.23	0.24
240	0.28	0.25	0.3	0.24	0.23	0.24

PaO2 (mmHg)

Post-surge	77				55	
Stable bas	59	76	64	58	58	55
0	60	66	56	62	61	63
60	51	76	58	57		75
120		57	57	57		62
180		67	63	56		75
240	47	65	75	67	49	70

PaCO2 (mmHg)

Post-surge	48				37	
Stable bas	47	38.2	37	35.6	35.8	37.7
0	46.3	42.5	40.1	32.9	28.5	40.1
60	48.9	40.2	37.8	18.6	30.4	36.3
120		38.6	49.5	18.9		42
180		44.9	44.9	19.1		40.4
240	57.5	42.9	40.1	20.1	41.3	37.3

OI

Post-surge	2.727273				4.14	
Stable bas	3.644068	2.875	3.414063	3.944828	4.005172	3.76363636
0	3.666667	3.380303	4.285714	3.820968	4.37377	3.5047619
60	4.264706	2.632895	4.224138	4.035088		2.848
120		3.903509	4.429825	4.824561		4.37419355
180		3.283582	3.690476	5.446429		2.912
240	5.123404	3.423077	3.88	3.689552	2.765823	4.28571429

AaDO2 (mmHg)

Post-surge	41.25					
Stable bas	60.5	40.24	53.74	54.36	61.24	61.865
5min post				na		
0	60.375	44.865	72.125	60.865	67.365	57.995
60	66.125	37.74	73	83.74		50.745
120		73	59.375	97.625		56.62
180		55.125	59.125	98.375		45.62
240	80.765	59.625	88.775	78.995	63.365	54.495

pH

Post-surge	7.35				7.48	
Stable bas	7.35	7.4	7.449	7.437	7.434	7.399
0	7.34	7.34	7.391	7.41	7.525	7.359
60	7.33	7.371	7.391	7.377	7.532	7.402
120		7.365	7.29	7.407		7.367
180		7.329	7.333	7.399		7.376
240	7.23	7.336	7.371	7.397	7.434	7.406

HCO3

Post-surge	26.4				27.6	
Stable bas	25.9	23.7	25.6	24	24	23.3
0	24.8	22.6	24.3	20.9	23.6	22.6
60	25.7	23.3	23	18.6	25.6	22.6
120		22.1	23.8	18.9		24.1
180		23.6	23.8	19.1		23.7
240	24.2	23	23.2	20.1	27.6	23.4

BE

Post-surge	1				4	
Stable bas	0	-1	2	0	0	-2
0	-1	-3	-1	-4	1	-3
60	0	-2	-2	-7	3	-2
120		-3	-3	-6		-1
180		-2	-2	-6		-2
240	-3	-3	-2	-5	3	-1

Hemoglobin (g/L)						
Post-surge	96	84	89	71	68	70
Stable bas	103	87	92	75	63	70
0	101	90	93	78	67	81
60	103	85	90	78	63	81
120		74	89	73	56	74
180		72	85	67	54	68
240	87	69	79	61	50	66

VT (ml/kg)						
Post-surge	14.7	14.68421	14.57895	13.16667	13.88235	12.4545455
Stable bas	14.7	14.63158	14.73684	14.22222	15.11765	13.5
0	14.7	12.73684	14.73684	14.27778	15.17647	13.0454545
60	14.7	14.63158	14.78947	14.22222	14	13.6818182
120	14.7	14.63158	15.73684	14.16667	13.94118	13.2727273
180	14.7	14.68421	15.73684	14.27778	13.88235	13.4545455
240	14.7	14.63158	14.89474	14.38889	13.88235	15.5

MV (ml/kg/min)						
Post-surgery						
Stable bas	735	731.5789	781.0526	782.2222	831.4706	769.5
0	735	636.8421	781.0526	785.2778	834.7059	717.5
60	735	804.7368	813.4211	782.2222	770	752.5
120	735	804.7368	865.5263	1119.167	766.7647	915.818182
180	735	807.6316	865.5263	1113.667	763.5294	740
240	735	804.7368	819.2105	791.3889	763.5294	806

Temp ©						
Post-surge	38.2	40	40.3	39.9	40	39.6
Stable bas	39.2	38.7	38.9	39.5	39.4	40
0	40.2	39.2	39.4	28.6	40.1	39.7
60	39.5	38.8	39.6	40.1	39.4	39.4
120	38.3	40.6	38.8	39.2	39.5	40.9
180	39.8	38.6	40	40	39.5	39.7
240	39.6	39.5	39.4	39.5	39.8	40

MILLAR

Tau (ms)

Stable bas	21.39	19.14	19.19	19.88	17.22	20.19
0	21.71	18.3	18.37	20.18	14.72	36
60	28.22	19.67	19.09	17.53	16.04	64.29
120	32.31	16.23	21.87	19.58	14.6	150.3
180	26.08	21.48	29.41	24.77	14.04	14.7
240	47.28	25.63	49.85	23.37	13.13	16.88

Stroke volume index (ml/kg)

Stable bas	1.074	0.348526	0.420737	0.307	0.309176	0.26304545
0	1.083333	0.321474	0.318368	0.418556	0.337588	0.18413636
60	1.061333	0.342105	0.301263	0.370389	0.345059	0.22318182
120	1.093333	0.338053	0.297737	0.425833	0.421353	0.22636364
180	1.1	0.360105	0.258632	0.438333	0.475824	0.44831818
240	1.141333	0.402421	0.336158	0.404	0.478706	0.36795455

Stroke work (mmHg*ml)

Stable bas	87.73	39.39	35.83	28.46	26.51	35.68
0	102.3	34.51	23.25	42.08	30.35	12.42
60	81.98	34.66	18.72	29.26	28.59	25.91
120	73.23	33.91	19.35	34.91	34.01	9.078
180	70.54	28.79	13.85	38.37	36.77	58.27
240	72.66	32.41	19.24	33.41	34.43	39.76

MILLAR DATA

Stroke work (% change from baseline)

Stable bas	1	1	1	1	1	1
0	0.71091	0.877411	0.64773	1.478566	1.143879	0.33944201
60	0.569979	0.879695	0.522417	1.02811	1.080979	0.70897155
120	0.508895	0.860914	0.539126	1.226634	1.280603	0.24909737
180	0.490202	0.730203	0.383737	1.348208	1.384934	1.58752735
240	0.504934	0.822081	0.53105	1.173928	1.518644	1.08561269

Stroke volume (% change from baseline)

Stable bas	1	1	1	1	1	1
0	0.730337	0.922241	0.756726	1.363175	1.094077	0.66430688
60	0.715955	0.981881	0.717557	1.207684	1.158065	0.80786961
120	0.737079	0.970104	0.708672	1.391809	1.364121	0.81988805
180	0.741573	1.033067	0.614066	1.430228	1.540849	1.62644057
240	0.769438	1.154009	0.790014	1.410656	1.658541	1.33141258

End-diastolic volume (% change from baseline)

Stable bas	1	1	1	1	1	1
0	1.103101	0.971233	0.929355	1.05574	0.990059	0.88747242
60	1.102263	0.971461	0.944493	1.070338	0.991612	0.9215494
120	1.097234	0.994064	0.951294	1.060053	0.994719	0.91002697
180	1.119866	0.97032	0.951733	1.042137	0.997204	0.91198823
240	1.082146	0.996119	0.976086	0.946251	0.999689	0.717823

End-diastolic pressure (% change from baseline)

Stable bas	1	1	1	1	1	1
0	1.116811	0.906493	0.643339	0.816642	0.818037	0.81000695
60	1.148382	1.000676	0.712851	0.793078	0.840183	0.94690757
120	1.183899	1.016064	0.744547	0.99908	0.926941	2.67129951
180	1.227309	1.09672	0.789581	1.17986	1.034475	0.8704656
240	1.163378	1.177038	0.871175	0.932622	1.155251	0.61626129

Ejection fraction (% change from baseline)

Stable bas	1	1	1	1	1	1
0	0.673246	0.954918	0.806864	1.298491	1.100429	0.75152749
60	0.661732	1.019809	0.761489	1.16825	1.16534	0.88594705
120	0.683114	0.986339	0.746364	1.29905	1.368647	0.99660557
180	0.676535	1.076503	0.647469	1.385131	1.543172	1.700611
240	0.717105	1.174863	0.802792	1.380101	1.657073	1.69110659

Max dP/dt (% change from baseline)

Stable bas	1	1	1	1	1	1
0	0.682666	0.737236	0.792331	0.606004	0.965578	0.61477987

60	0.625617	0.586438	0.812691	0.457932	0.750263	0.56199461
120	0.493692	0.657402	0.972514	0.463306	0.775553	0.71473495
180	0.497532	0.390893	0.801493	0.55467	0.770636	0.67610063
240	0.546901	0.28839	0.859518	0.691438	0.814542	0.5384097

Min dP/dt (% change from baseline)

Stable bas	1	1	1	1	1	1
0	0.949637	1.321311	0.672376	1.02963	1.009282	0.65930345
60	0.758522	0.841967	0.43351	1.197821	0.756396	0.28023218
120	0.572245	0.967541	0.398941	0.983007	0.781752	0.2117059
180	0.620189	0.549508	0.353005	0.75817	0.834956	0.86375363
240	0.519463	0.61082	0.359701	0.708061	0.736699	0.63608513

Tau (% change from baseline)

Stable bas	1	1	1	1	1	1
0	0.737182	0.956113	0.957269	1.011529	0.855148	1.86721992
60	0.958913	1.028213	0.993747	0.877694	0.927283	3.33143154
120	1.096435	0.846917	1.132882	0.980451	0.850494	7.74896266
180	0.885569	1.12278	1.544033	1.226566	0.817336	0.7629668
240	1.598981	1.356322	2.637832	1.07218	0.745782	0.87966805
