# Supplementary material for: Hemodynamic effects of high frequency oscillatory ventilation with volume guarantee in a piglet model of respiratory distress syndrome
Source: PLoS One. 2021 Feb 16;16(2):e0246996. doi: 10.1371/journal.pone.0246996 (PMC7886162; doi:10.1371/journal.pone.0246996)
Supplement: S1 File — (PDF) [file pone.0246996.s005.pdf]

S1 Table – Left ventricle measures (fraction of baseline or absolute value) from Millar® catheter based on time and mode of ventilation, shown as mean (one standard deviation). No significant differences.

EF = ejection fraction, SW = stroke work, dP/dt = measure of contractility, EDV = end-diastolic volume, EDP = end-diastolic pressure

	**Time point (minutes)**				
**Parameter and mode**	**0**	**60**	**120**	**180**	**240**
EF (fraction of baseline)					
Sham	0.93 (0.24)	0.94 (0.21)	1.01 (0.28)	1.17 (0.45)	1.24 (0.42)
HFOV	0.87 (0.18)	0.88 (0.26)	0.84 (0.25)	0.97 (0.25)	1.06 (0.24)
HFOV+VG	1.00 (0.10)	1.10 (0.29)	1.04 (0.13)	1.14 (0.36)	0.95 (0.21)
SW (mmHg*ml)					
Sham	40.8 (31.8)	36.5 (22.9)	34.1 (21.8)	41.1 (20.4)	38.7 (18.0)
HFOV	34.4 (11.4)	34.8 (19.5)	29.7 (12.5)	37.4 (12.0)	41.3 (18.1)
HFOV+VG	36.7 (17.6)	29.9 (15.9)	27.9 (17.6)	27.3 (20.4)	28.2 (21.6)
dP/dt min (fraction of baseline)					
Sham	0.94 (0.25)	0.71 (0.32)	0.65 (0.31)	0.66 (0.19)	0.60 (0.14)

HFOV	0.80 (0.25)	0.95 (0.34)	0.85 (0.36)	0.83 (0.40)	0.64 (0.20)
HFOV+VG	0.90 (0.33)	0.89 (0.40)	0.83 (0.38)	0.85 (0.58)	0.78 (0.72)
EDV (fraction of baseline)					
Sham	0.99 (0.08)	1.00 (0.07)	1.00 (0.07)	1.00 (0.07)	0.95 (0.12)
HFOV	0.91 (0.05)	0.90 (0.09)	0.91 (0.09)	0.93 (0.11)	0.94 (0.11)
HFOV+VG	0.98 (0.06)	0.95 (0.08)	0.95 (0.09)	0.95 (0.11)	0.96 (0.12)
EDP (fraction of baseline)					
Sham	0.85 (0.16)	0.91 (0.16)	1.26 (0.71)	1.03 (0.17)	0.99 (0.22)
HFOV	1.04 (0.17)	0.91 (0.29)	0.88 (0.22)	0.99 (0.30)	0.96 (0.29)
HFOV+VG	1.18 (0.13)	1.39 (0.90)	1.09 (0.26)	1.34 (0.72)	1.20 (0.24)

baseline = before lavage
HFOV = high frequency oscillatory ventilation
VG = volume guarantee

S2 Table – Tissue lactate and GSSG/GSH for left ventricle and brain based on mode of ventilation, shown as mean (one standard deviation). No significant differences.

	**HFOV**	**HFOV+VG**	**Sham**
**Tissue lactate (mmol/mg)**			
Left ventricle	8.5×10^{-4} (2.5×10^{-4})	11×10^{-4} (5.7×10^{-4})	7.6×10^{-4} (2.6×10^{-4})
Brain	7.0×10^{-4} (1.7×10^{-4})	7.3×10^{-4} (1.8×10^{-4})	7.2×10^{-4}(0.98×10^{-4})
**GSSG/GSH (ratio)**			
Left ventricle	0.03 (0.02)	0.02 (0.01)	0.02 (0.01)
Brain	0.12 (0.06)	0.11 (0.02)	0.09 (0.01)

HFOV = high frequency oscillatory ventilation
VG = volume guarantee
