# Supplementary material for: Hemodynamic effects of high frequency oscillatory ventilation with volume guarantee in a piglet model of respiratory distress syndrome
Source: PLoS One. 2021 Feb 16;16(2):e0246996. doi: 10.1371/journal.pone.0246996 (PMC7886162; doi:10.1371/journal.pone.0246996)
Supplement: Attachment — Submitted filename: Rebuttal - HFOV+VG hemodynamics in RDS piglets.docx [file pone.0246996.s006.docx]

Responses to Reviewers’ and Academic Editor’s Comments

We are grateful to the insightful comments of both reviewers and the academic editor. The reviewers also complimented our interesting observations and their contribution to the literature. We believe that we have adequately addressed these comments in the revised manuscript and improved the scientific merit of the submission, which becomes a technically sound piece of scientific research with data that supports the conclusions.

Specific responses to each comment:

**Reviewer #1:**

1. **HFOV+VG vs. HFOV and carotid artery flow index and NIRS:** Thank you for the comment and we agree that our speculation that the reason for the change in carotid blood flow was related to the fluctuating amplitude during HFOV+VG was weak as the reviewer cited that delta P generated by the ventilator was not transmitted into the airways in an experimental model (Kamitsuka et al 1990). We included this in our discussion on page 19 (line 374-378). We also admitted that there was no effect of HFOV+VG on the CO and cardiac parameters measured by Millar catheter when compared with HFOV. However, we did not study the mechanistic pathway between fluctuating amplitude and cerebral perfusion in the absence of any significant effect on the intrathoracic pressure, CO and other cardiac parameters.
2. **Amplitude and Tidal Volume:** As the point regarding the amplitude and tidal volume was commented by both reviewers, we further examined the changes in amplitude and tidal volume during HFOV+VG and HFOV modes in these animals after saline lavage. We found that the amplitude was higher in the HFOV+VG group than that of the HFOV group whereas the tidal volume was not different between groups. We are not certain about the relevance of this observation. The results (P.13, line 240-242 and 250-253, Fig.3) and discussion (P.19 line 360-365) had been added in the revised manuscript. This point is important and should generate further research to understand the relationship between HFOV+VG and cerebral perfusion, which was under-studied.
3. **Animal model:** The lack of lung recruitment maneuver after saline lavage is a limitation of our study although this clinical practice might further destabilize our critically sick animals. We have included this in the methods (P.7, line 146-147) and discussion on page 22 (line 428-437).
4. **Oscillatory mechanism:** The discussion of oscillatory mechanisms of the Fabian HFO vs. Babylog VN500 was also included on P.23 line 453-457. We agree that this is a main variable that may be different during HFOV with the VG mode.
5. **Results:** Thanks to this reviewer who pointed out the 1-2 outliers in the groups regarding the NIRS, CO, carotid blood flow and arterial pH. Although the increased variance precluded us from identifying significant differences between groups, passing the normal distribution and equal variance pre-priori testing indicated the appropriate use of statistical analyses. The reviewer also graciously explained that DCO_2_ is superior to MV as a measure of ventilation in HFOV, so we have added the DCO_2_ data to the supplemental files immediately below the MV data (S1_Table, S2_Table).
6. **Final comments:** We are grateful to this reviewer who commented that this is an interesting study exploring the potential different effect of the VG mode when used in combination with HFOV instead of not using it. S/he highlighted the key concept that changes in the lung and patient condition during ventilation may result in fluctuation in delta P to maintain the set tidal volume in the VG mode and therefore may account for the potential differences in the observation of hemodynamics. If the patient’s lung is stable, then both modes will work equally at similar tidal volumes. This important point has been highlighted in the revised manuscript on page 19 line 379-381.

**Reviewer #2:**

1. **Open lung strategy is the key element of lung protective strategies:** We are thankful for this insightful comment. As indicated by reviewer #1, this is a limitation regarding the translational value of this animal model. We adopted the model with modifications in newborn piglets. There were a few factors that limited the inclusion of lung recruitment maneuvers. These factors included the increasing saline lavage duration, lengthy procedure and interactive compromise of the cardiovascular system which might not tolerate recruitment maneuvers. Further improvements in the model will be needed. This limitation has been added to the revised version on page 22 (line 428-437). We also discussed the confounding effects of lung recruitment maneuvers on temporarily increased transpulmonary pressure, ventilator parameters including lowered MAP, amplitude and tidal volume, and pO2 and pCO2, which might affect the systemic and regional hemodynamics. The interesting speculation of effects of open lung strategy depending on HFOV mode was also included.
2. **Extrapolation of results to newborn babies with RDS:** We thank this reviewer who pointed out the limitation that clinically newborn babies with RDS have received surfactant and usually require the use of inotropic agents. This limitation has been added to the discussion on page 21-22 line 425-427.
3. **Relationship between HFOV on systemic hemodynamics:** We agree with this reviewer that MAP, not tidal volume/delta P, is the strongest effector of intrathoracic pressure (P.19, line 376-378). This supports our observation that the CO was not affected by the mode of HFOV as both groups had similar MAP. As this reviewer indicated, the increased hypotension and lower perfusion findings with HFOV-VG mode compared to HFOV mode (using similar MAP levels) are interesting and require further investigation. The relationship between amplitude and intrathoracic pressure and MAP are interesting and need further investigation if this is the etiology for worsened hemodynamics (P.19, line 375-376).

**Academic Editor:**

1. **Lung recruitment:** This has been added in the methods (P.7, line 146-147) and discussion (P.22, line 428-437).
2. **The relationship between cardiac output, transmural pressure, delta P (amplitude) and mean airway pressure:** As mentioned above that both reviewers had different opinions on the relationship, we have added the complex relationship and the additional observations on the amplitude and tidal volume during HFOV+VG and HFOV in the discussion (P.19, line 372-381).
3. **Sham blood pressure low at 240min**: Table 3 shows the mean blood pressure of the sham-operated piglets at 240min was 43mmHg, which was lower than baseline but not significantly different between the experimental groups. This could be related to the effect of anesthetic and surgical interventions in this model.
4. **Anesthesia:** The editor’s comment that anesthesia could be a factor relating to systemic and regional measurements is an excellent one. Although similar anesthetic/analgesic dose infusion ranges were used for each piglet we did not record the precise amounts given so are unable to rule it out as a factor. This has been added to the limitations on page 21, line 418-421. The inclusion of sham-operated piglets could help interpret the hemodynamic effects of two HFOV modes. Nevertheless, all animals were closely monitored throughout the experiments with adjustment of anesthetic and analgesic medications according to their neurological status. This detail has been added to the methods on page 6, line 105-106.
5. **CrSO_2_ graph Figure 3B:** We thank the editor for the observation regarding the discrepancy between absolute values in the table and percent change in the figure. The graph has been changed accordingly.
6. **Data not shown**: An additional supplemental figure has been added (S4_Table) which contains the previously mentioned ‘data not shown’ in mean ± standard deviation format, which includes Millar® catheter and tissue data.

Sincerely,

Jag Bhogal and Po-Yin Cheung
